# Cognitive function and quantitative electroencephalogram analysis in subjects recovered from COVID-19 infection

**DOI:** 10.1186/s12883-023-03518-7

**Published:** 2024-02-10

**Authors:** Manal M. Gaber, Hanan Hosny, Mona Hussein, Mona A. Ashmawy, Rehab Magdy

**Affiliations:** 1 Clinical Neurophysiology Department, Neuro Diagnostic Research Center (NDRC), Beni-Suef University, Beni-Suef, Egypt; 2 Department of Neurology, Faculty of Medicine, Beni-Suef University, Beni-Suef, Egypt; 3 Department of Neurology, Faculty of Medicine, Cairo University, Cairo, Egypt

**Keywords:** Post-COVID-19, Cognition, QEEG, PALT, PASAT

## Abstract

**Background & Objectives:**

Objective assessment of post-COVID-19 cognitive dysfunction is highly warranted. This study aimed to evaluate the cognitive dysfunction of COVID-19 survivors with cognitive complaints, both clinically and neurophysiologically, using Quantitative Electroencephalogram (QEEG).

**Methods:**

This case–control study was conducted on 50 recovered subjects from COVID-19 infection with cognitive complaints and 50 age, sex, and educational-matched healthy controls. Both groups were subjected to the following neurocognitive tests: Paired associate learning Test (PALT) and Paced Auditory Serial Addition Test (PASAT). The neurophysiological assessment was also done for both groups using QEEG.

**Results:**

COVID-19 survivors had significantly lower PALT scores than controls (*P* < 0.001). QEEG analysis found significantly higher levels of Theta / Beta ratio in both central and parietal areas in patients than in the controls (*P* < 0.001 for each). The interhemispheric coherence for the frontal, central, and parietal regions was also significantly lower in patients than in the control group regarding alpha and beta bands. There were statistically significant lower scores of PALT and PASAT among cases with severe COVID-19 infection (*P* = *0.011, 0.005*, respectively) and those who needed oxygen support (*P* = 0.04, 0.01, respectively). On the other hand, a statistically significantly lower mean of frontal alpha inter-hemispheric coherence among patients with severe COVID-19 infection (*P* = 0.01) and those needing mechanical ventilation support (*P* = 0.04).

**Conclusion:**

Episodic memory deficit is evident in COVID-19 survivors with subjective cognitive complaints accompanied by lower inter-hemispheric coherence in frontal regions. These clinical and neurophysiological changes are associated with hypoxia and COVID-19 severity.

## Introduction

Long-term neurological sequelae of Coronavirus disease (COVID-19) have become a significant public health issue worldwide, including chronic fatigue [1], sleep disturbance [2], cognitive dysfunction [3], migraine-like headaches [4], and peripheral nervous system involvement [5]. Hypoxia, systemic inflammation with cytokine release, direct viral brain invasion, blood–brain barrier dysfunction, and cerebral ischemia are possible underlying mechanisms of how the SARS-CoV-2 virus can induce various neurological sequelae [6].

Post-COVID-19 cognitive impairment has been widely described in previous reports using various neuropsychological assessments in the form of attention, information processing speed, memory, and executive function [7–13]. Quantitative electroencephalography (QEEG) can precisely evaluate cortical function and functional brain connectivity. Thus, QEEG is a valuable biomarker broadly used in various cognitive disorders in clinical and research settings [14, 15].

In the post-COVID-19 cognitive dysfunction research field, QEEG has been used by G Cecchetti et al. [16], who found a decrease in individual alpha frequency and an increase in cortical current source densities (CSD) at the delta band of COVID-19 survivors. By the 10-month follow-up, the lower delta band at baseline could predict worse cognitive performance. However, other quantitative methods, including power ratios, coherence, and connectivity analyses, were not employed.

One of the power ratios commonly used in variable neuropsychiatric disorders is theta/ Beta ratio, recently used as a marker of cognitive processing capacity [17]. E Pasini et al. [18] found that EEG abnormalities in COVID-19 encephalopathy affect the frontoparietal connectivity that is supposed to be a potential biomarker of post-COVID-19 cognitive dysfunction.

Moreover, a previously published PET study about post-COVID-19 cognitive dysfunction demonstrated that the frontoparietal cortex is the most affected brain region [19]. Accordingly, we wanted to study the neurophysiological base of such region by QEEG analysis in relation to cognitive tasks mediated by this region. Hence, we hypothesized that COVID-19 infection may affect processing speed and episodic memory associated with a higher Theta / Beta ratio and lowered coherence in the fronto-parital region.

This work aimed to assess the cognitive function of COVID-19 survivors with subjective cognitive complaints, both clinically and neurophysiologically, using QEEG, in particular theta/ Beta ratio and interhemispheric coherence in the fronto-parital region.

## Methods

### Participants and eligibility criteria

This case–control study was conducted in the period between April 2022 and December 2022. Fifty COVID-19 survivors seeking medical help for a subjective cognitive concern were recruited from Neurology Clinic, Beni-Suef University Hospital (patients group). All in the patients' group must have a documented positive SARS-CoV-2 polymerase chain reaction (PCR) on a nasopharyngeal sample, upon which a diagnosis of COVID-19 is made.

Another 50 age, sex, and educational-matched healthy volunteers were selected randomly from the normal database pool registered in our unit to be used for research purposes. Clinical and neurophysiological data were extracted from the database if conducted before the beginning of the pandemic to ensure the complete exclusion of individuals possibly infected with COVID-19 from the control group.

All included subjects should have at least 12 years of education. All of them underwent brain MRI, which must be normal for eligibility in this study. Exclusion criteria for both groups included illiteracy, major depressive disorder based on DSM-IV criteria, medical, neurological disease (e.g., diabetes, stroke, multiple sclerosis), or substances that may affect cognitive functions (e.g., opioids and cannabis) and severe physical or auditory impairment that may affect the ability to perform cognitive testing.

### Measures

The included patients were subjected to the following:

1. History taking regarding demographics, comorbidities, COVID-19 symptomatology, duration of illness, and need for respiratory support, either oxygen or mechanical ventilation. All patients were requested to send their chest CT to allow for assessment of pulmonary involvement by The coronavirus disease 2019 (COVID-19) Reporting and Data System (CO-RADS), which ranged from 1 to 5 [20].
2. The severity of infection was defined according to WHO classification [21]. The mild state was characterized by typical symptoms without evidence of viral pneumonia or hypoxia. At the same time, moderate or severe cases were identified if there was any clinical and radiological evidence of pneumonia. In moderate infection, patients had to have SpO2 ≥ 90% on room air, while one of the following was required to define the severe cases: respiratory rate > 30 breaths/min; severe respiratory distress; or SpO2 < 90% on room air.
3. Cognitive assessment:

The main complaints of the included patients were subjective attention and memory deficits. Accordingly, we chose cognitive tests that targeted these domains. The cognitive function of the included subjects in both groups was assessed using the following cognitive tests:

-
  a Paired Associate Learning test (PALT) (Spaan P et al., 2005) [22]
- This test was used to assess verbal learning and memory. The examiner says ten associated pairs in front of the candidate (six compatible semantically related pairs and four incompatible semantically unrelated pairs). After one minute, the candidate is given the pairs' first word and asked to recall the second word. The test is repeated three times. Each correct compatible pair takes a score of 0.5, while each correct incompatible pair takes a score of 1. The total score ranges from 0 to 21.
  b Paced Auditory Serial Addition Test (PASAT): (Gronwall D M 1977) [23]

It was used for the assessment of attention and auditory working memory. In this test, 61 single-digit numbers are spoken on an audiotape, one every 3 s. The subject is asked to add each number to the one immediately preceding it and not to give a running total. The subject has to report the sum orally. The total score is the sum of correct responses ranging from 0 to 60.

4) Neurophysiological assessment

The patients and controls underwent QEEG using a Galileo NT PMS device made in Italy at the Neurodiagnostic Research Center (NDRC), Beni-Suef University Hospital. Then The raw EEG data are further quantified using NeuroGuide Software.

(A) EEG Recording:

Nineteen gold disc electrodes were placed on the subject’s scalp using electrode paste; according to the international 10/20 system of electrode placement at electrode locations FP1, FP2, F7, F3, FZ, F4, F8, T3, C3, CZ, C4, T4, T5, P3, PZ, P4, T6, O1, and O2 with reference and ground electrodes placed at the forehead. The impedances of the electrodes were always below 5 kohms. Raw EEG signals were recorded using a Galelio EEG device with a 1–70 Hz frequency band. During the twenty-minute session of EEG recording, the subject was lying supine during a state of relaxed wakefulness in a silent environment. An EEG technician followed the recording to monitor the signal quality, minimize eye and muscle artefacts, and ensure the wakeful state. The recording alternates between eye-closed and eye-opened conditions (ten minutes each).

(B) Mini-QEEG Processing:

For EEG power analyses, a total of two minutes of artefact-free EEG data was selected from the eyes-opened conditions. Epochs showing findings of a drowsy state or containing eye movements, blinking, or muscle activity were carefully screened out by visual inspection. The Montage used was linked ear Montage.

Power spectral analysis was performed using fast Fourier transformation (FFT) for different frequency bands, and frequency spectra were averaged across all selected epochs at each recording site to obtain the absolute band power and the power ratio. The frequency bands were as follows: Delta (1–4 Hz), Theta (4–8 Hz), Alpha (8- 13 Hz), and Beta (13–30 Hz). The Selected electrodes in this mini-QEEG were frontal for F3 and F4, Central for C3 and C4, and Parietal for P3 and P4. Interhemispheric coherence was measured between right and left frontal (F3-F4), Central (C3- C4), and parietal (P3-P4) electrodes.

### Ethical statement

All the individuals included in the study were informed about the procedures, and all agreed to participate. The participants were informed of their rights to refuse participation or withdraw from the study without giving reasons. All information was treated with confidentiality. The informed consent was obtained from all the participants in this study. All the methods were performed in accordance with the relevant guidelines and regulations.

Before starting the research study, approval was obtained from the ethical approval of the faculty of medicine, Beni-Suef University Research Ethical Committee (REC). The ethical approval number is FMBSUREC/08052022/Soliman.

### Statistical methods

Data analysis was performed using the Statistical Package of Social Science (SPSS) software version 22 in Windows 7 (SPSS Inc., Chicago, IL, USA). Nominal data such as sex, COVID-19 grade and severity, and need for oxygen or mechanical ventilation were expressed as number and percent. Normally distributed quantitative variables such as age and education were expressed as mean and standard deviation (SD), whereas quantitative variables not normally distributed, such as neurocognitive tests and QEEG parameters, were expressed as median and interquartile range (IQR). The chi-square test was used to compare between patients and control groups in nominal variables. Independent samples t-test was used to compare between two independent groups in normally distributed variables, while the Mann–Whitney test was used to compare between two independent groups in non-normally distributed variables. Kruskal–Wallis test was used to compare three independent groups in non-normally distributed variables. Multivariate linear regression analysis was done to determine predictors of cognitive performance among COVID-19 survivors. The *P*-value ≤ 0.05 was considered statistically significant. All tests were two-tailed.

## Results

This case–control study evaluated 50 COVID-19 survivors with subjective cognitive complaints and 50 healthy controls.

Among COVID-19 cases, the mean duration of illness was 19.9 ± 12.4 days, while the mean recovery duration was 6.04 ± 3.3 months. Twenty-one patients (42%) were categorized as having mild COVID-19 infection, 25 (50%) had moderate, and only four patients (8%) had a severe infection.

By CORAD categorization, most of the patients (27, 54%) had grade IV category, followed by grade V in 22 (44%) and grade III in one patient (2%). However, Oxygen support was needed in 25 patients (50%), while mechanical ventilation was required in only 4 cases (8%). Only five cases needed ICU admission.

Both groups were matched in age, sex, and educational years. Patients had significantly lower scores in PALT than healthy controls. However, PASAT scores did not significantly differ by applying the Benjamini–Hochberg FDR correction (Table 1).

**Table 1:** Demographics and neurocognitive assessment in COVID-19 survivors versus the healthy control

Age (Mean ± SD)		Patients (*n* = 50)	Control (*n* = 50)	*P*-value
		36.3 ± 8.1	33.2 ± 8.9	0.07
Gender	Male	26 (52%)	31 (62%)	0.4
	Female	24 (48%)	19 (38%)	
Education		13.03 ± 2.75	14.08 ± 2.42	0.074
PALT (Median /IQR)		12/15	16.7/11.5	< 0.001
PASAT (Median /IQR)		51/7	40/48	0.04*

*PALT* Paired associate learning Test, *PASAT* Paced Auditory Serial Addition Test

^*^did not remain statistically significant after applying the Benjamini–Hochberg FDR correction

**Fig. 1:**
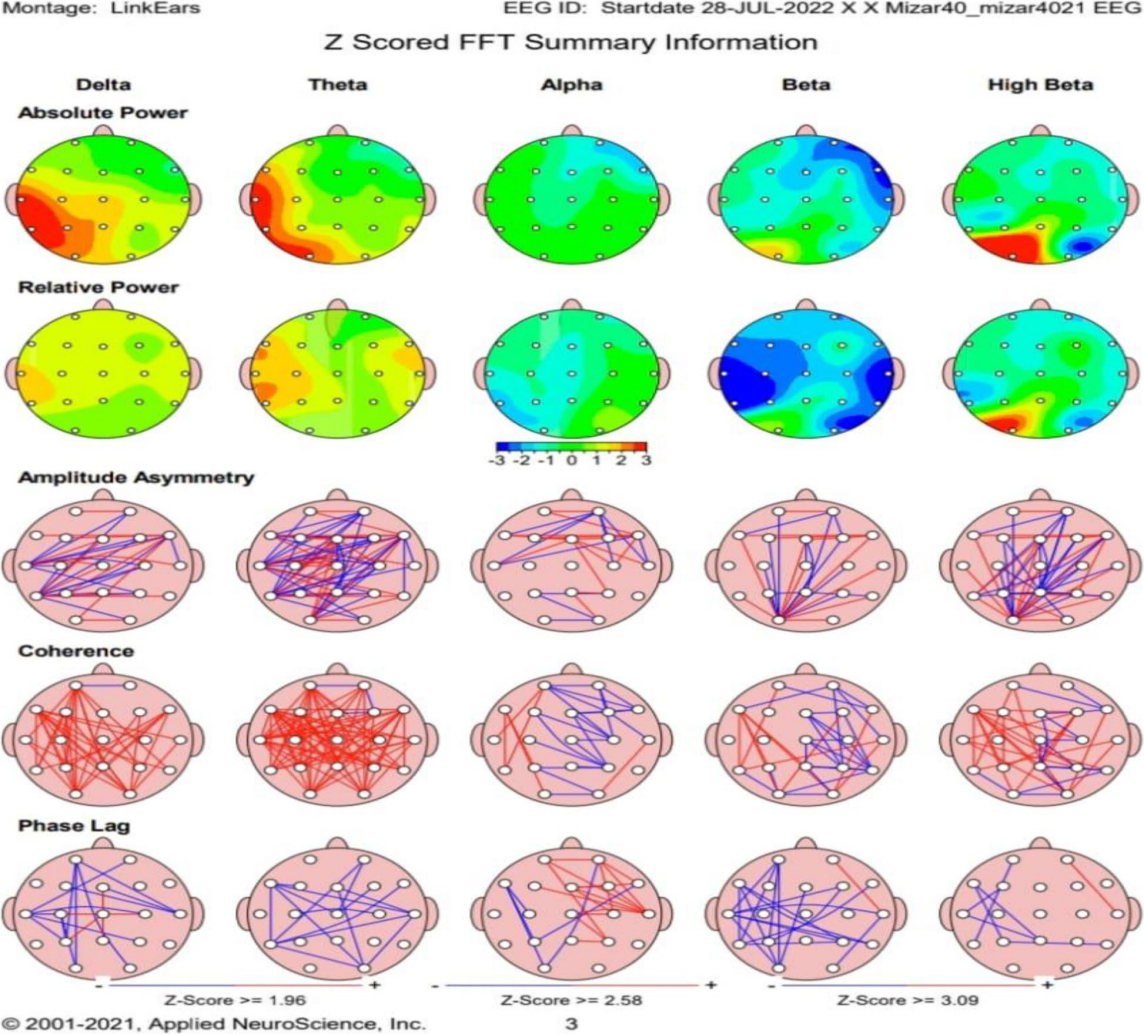
A case of female patient 33 years old, the EEG recorded in open eye condition, as seen in there is relatively high theta absolute power in the parietal region with relatively reduced alpha absolute power in the fronto central areas, together with hypocoherence as regard alpha band in frontal and central areas and hypocoherence as regard beta band in frontal, central and parietal areas. Also there's increase in theta power with decrease in beta power results in high Theta beta ratio

QEEG analysis found that the patients' group had a statistically significant higher absolute theta power value in frontal, central, and parietal regions and a lower level of alpha absolute power in central and parietal regions (Table 2). In addition, a statistically significant higher level of Theta / Beta ratio in both central and parietal areas was found in patients than in the control group. The interhemispheric coherence for the frontal, central, and parietal regions was significantly lower in patients than in the control group, evident in both alpha and beta bands (Table 2). Figure 1 illustrates QEEG map of one othe included patients.

**Table 2:** Quantitative EEG analysis in COVID-19 survivors versus the healthy control

			Patients (*n* = 50) Median /IQR	Control (*n* = 50) Median /IQR	*P*-value
Frontal Absolute power		Frontal Theta	22.2/17.8	7.6/5.6	< 0.001*
		Frontal Alpha	6.1/4.8	6.7/6.4	0.3
Central Absolute power		Central Theta	21.3/15.5	7.8/4.2	< 0.001*
		Central Alpha	6.5/8.1	8.9/7.7	0.01*
Parietal Absolute power		Parietal Theta	21.3/21.2	8.6/5.6	< 0.001*
		Parietal Alpha P	10.5/10.02	16.5/15.7	< 0.001*
Power ratio (theta/beta ratio)		C3	1.7/2.3	0.89/0.46	< 0.001*
		C4	1.5/2.2	0.96/0.38	< 0.001*
		P3	1.7/1.9	0.86/0.45	< 0.001*
		P4	1.4/2.2	0.92/0.7	< 0.001*
Interhemispheric coherence	F3/F4	Alpha	20.2/14.03	54.7/35.5	< 0.001*
		Beta	14.1/20.8	28.8/30.4	< 0.001*
	C3/C4	Alpha	23.6/26.03	25/22.2	0.02*
		Beta	19.9/22.7	26.1/25.9	0.03*
	P3/P4	Alpha	22.7/17.3	38.9/12.2	< 0.001*
		Beta	14.1/23.5	31.9/22.1	< 0.001*

^*^significance difference with *P*-value < 0.05

There was a statistically significant lower means of PALT and PASAT scores among cases with severe COVID-19 infection and those who needed oxygen support. Additionally, patients with category CORAD 5 had a significantly lower mean of PALT than those with category IV (Table 3).

**Table 3:** Neurocognitive assessment in COVID-19 survivors in relation to CORADS grading, COVID-19 severity, and need for respiratory support

Variables		Neurocognitive tests Median /IQR	
		PALT	PASAT
CORADS grade			
III		14/0	58/0
IV		13/12	36/50
V		11/12	21/50
*P*-value		0.03*	0.06
COVID-19 severity			
Mild		14/12	46/51
Moderate		11/13	19/50
Severe		8.3/12	11.5/46
*P*-value		0.011*	0.005*
Respiratory support			
Oxygen	No	13/15	41/51
	Yes	11/13	19/50
*P*-value		0.04*	0.01*
Mechanical ventilation	No	12/15	31.5/51
	Yes	8.3/12	11.5/46
*P*-value		0.1	0.2

*PALT* Paired associate learning Test, *PASAT* Paced Auditory Serial Addition Test, *CO-RADS* The coronavirus disease 2019-Reporting and Data System

^*^significance difference with *P*-value < 0.05

On the other hand, there was no statistically significant difference in levels of QEEG absolute power or power ratio values in relation to CORADS grading, COVID-19 severity, need for Oxygen support, or mechanical ventilation (Tables 4 & 5). However, a statistically significantly lower mean of frontal alpha inter-hemispheric coherence among patients with CORAD 5 degree, severe COVID-19 infection, and those needing mechanical ventilation support (Table 6).

**Table 4:** QEEG absolute power among COVID-19 survivors in relation to CORADS grading, COVID-19 severity, and need for respiratory support

Variables		QEEG absolute power parameters (Median /IQR)					
		Frontal		Central		Parietal	
		Theta	Alpha	Theta	Alpha	Theta	Alpha
CORADs grade							
III		24.28/0	11.16/0	19.93/0	12.23/0	41.8/0	17.17/0
IV		22.5/85.7	6.1/31.7	16.7/65.7	6.1/20.7	18.8/84.9	9.4/23.5
V		21.9/50.7	5.85/21.8	23.02/80.2	7.5/25.2	24.2/85.2	11.7/23.6
*P*-value		0.8	0.9	0.4	0.3	0.4	0.6
COVID-19 severity							
Mild		19.9/36.8	7/31.7	19.9/31.3	6.5/12.02	19.14/35.5	10.24/23.5
Moderate		23.7/80.3	5.9/21.8	21.3/80.2	7.4/24.5	24.5/85.2	11.3/23.1
Severe		21.9/36.3	5.7/5.2	23.02/34.9	5.9/7.9	21.4/47.6	9.9/9.8
*P*-value		0.4	0.7	0.2	0.4	0.8	0.8
Respiratory support							
Oxygen	No	21.8/49.3	7/31.6	19.9/41.9	6.5/12.02	19.1/53.1	10.2/23.5
	Yes	23.7/80.3	5.8/21.8	21.3/80.2	7.3/24.5	24.5/85.2	11.3/23.1
*P*-value		0.3	0.9	0.1	0.2	0.5	0.6
Mechanical ventilation	No	22.5/85.7	6.1/31.7	20.6/81.4	6.9/25.5	19.5/87.8	10.5/24.3
	Yes	21.9/36.3	5.7/5.1	23.02/34.8	5.9/7.9	21.4/47.6	9.9/9.7
*P*-value		0.6	0.4	0.8	0.5	0.8	0.5

*CO-RADS* The coronavirus disease 2019-Reporting and Data System, *QEEG* Quantitative electroencephalography

**Table 5:** Post-COVID-19 power ratios in relation to CORADS grading, COVID-19 severity, and need for respiratory support

Variables		QEEG power ratio (T/B ratio) parameters Median /IQR			
		C3	P3	C4	P4
CORADs grade					
III		1.02/0	1.3/0	1.5/0	2.1/0
IV		1.7/7.2	6.2/6.2	1.4/7.7	1.6/6.2
V		1.66/7.8	1.77/9.3	1.68/8.1	1.28/11.2
*P*-value		0.6	0.7	0.6	0.8
COVID-19 severity					
Mild		1.7/7.2	1.5/6.2	1.4/7.7	1.6/6.2
Moderate		1.4/7.9	1.7/9.3	1.5/8.3	1.2/11.2
Severe		2.5/4.8	2.4/7.2	1.9/5.2	2.3/6.7
*P*-value		0.3	0.3	0.3	0.4
Respiratory support					
Oxygen	No	1.9/7.2	1.5/7.8	1.8/7.7	1.9/7.4
	Yes	1.4/7.9	1.7/9.3	1.5/8.3	1.2/11.2
*P*-value		0.9	0.8	0.7	0.1
Mechanical ventilation	No	1.5/8.4	1.6/9.4	1.4/8.6	1.3/11.5
	Yes	2.5/4.7	2.4/7.2	1.9/5.2	2.3/6.7
*P*-value		0.1	0.2	0.1	0.2

*CO-RADS* The coronavirus disease 2019-Reporting and Data System, *QEEG* Quantitative electroencephalography

**Table 6:** Post-COVID-19 inter-hemispheric coherence values in relation to CORADS grading, COVID-19 severity, and need for respiratory support

Variables		QEEG inter-hemispheric coherence parameters (Median /IQR)					
		F3/F4		C3/C4		P3/P4	
		Alpha	Beta	Alpha	Beta	Alpha	Beta
CORADs grade							
III		33.5/0	36.8/0	34.2/0	30.7/0	39.3/0	27.1/0
IV		24.7/55.6	19.6/61.4	23.7/38.5	12.9/44.8	23.6/77.3	14.1/42.9
V		14.02/32.6	12.8/34.1	22.2/34.5	21.1/35.9	21.8/51.6	16.01/31.3
*P*-value		0.001*	0.2	0.2	0.4	0.2	0.6
COVID-19 severity							
Mild		24.7/55.6	23.2/61.4	23.6/37.9	12.9/44.9	21.1/68.3	10.3/42.9
Moderate		17.8/33.7	14.3/43.6	23.5/38.5	23.5/39.9	24.1/77.6	22.3/34.5
Severe		12.7/17.5	6.7/11.9	19.7/28.8	18.5/26.2	17.8/12.2	15.9/18.1
*P*-value		0.01*	0.5	0.7	0.6	0.6	0.8
Respiratory support							
Oxygen	No	23.9/58.5	12.3/61.4	23.6/38.5	12.9/44.9	21.1/68.3	11.2/42.9
	Yes	17.8/33.7	14.3/43.6	23.5/38.5	23.5/39.9	24.1/77.6	22.4/34.6
*P*-value		0.1	0.9	0.8	0.3	0.9	0.6
Mechanical ventilation	No	21.5/59.1	14.6/61.4	23.5/38.5	19.9/44.8	23.6/77.6	14.1/42.9
	Yes	12.6/17.5	9.6/11.9	19.7/28.8	18.5/26.3	17.8/12.2	15.9/18.1
*P*-value		0.04*	0.3	0.5	0.6	0.3	0.5

*CO-RADS* The coronavirus disease 2019-Reporting and Data System, *QEEG* Quantitative electroencephalography

^*^significance difference with *P*-value < 0.05

Multivariate linear regression models were conducted to identify predictors of cognitive performance among COVID-19 survivors. It was found that age and severity of COVID-19 infection were predictors of performance on PASAT, while age was the only predictor of performance on PALT (Table 7).

**Table 7:** Predictors of cognitive performance among COVID-19 surviviors by multivariate linear regression analysis

	PALT				PASAT			
	B	Standardized Coefficients Beta	t	*P*-value	B	Standardized Coefficients Beta	t	*P*-value
(Constant)	23.748		5.231	0.000	55.385		2.412	0.020
Age	-0.182	-0.408	-3.340	**0.002**	-0.772	-0.348	-2.805	**0.007**
Severity of COVID-19	-1.623	-0.281	-1.548	0.129	-11.572	-0.402	-2.181	**0.034**
CORADs grade	-0.334	-0.050	-0.265	0.792	6.035	0.180	0.949	0.348
Oxygen support	-1.099	-0.153	-1.022	0.312	-9.917	-0.278	-1.823	0.075

*CO-RADS* The coronavirus disease 2019-Reporting and Data System, *PALT* Paired associate learning Test, *PASAT* Paced Auditory Serial Addition Test

^*^significance difference with *P*-value < 0.05

## Discussion

Tremendous scientific efforts related to the long-term effect of COVID-19 infection on mental health are mounting. The present study explored the pattern of cognitive involvement of 50 COVID-19 survivors with subjective cognitive complaints demonstrated by verbal episodic memory deficits.

R Ferrucci and M Dini [24] described a similar cognitive profile among COVID-19 survivors one year after hospital discharge. In 2021, A Søraas et al. [25] demonstrated a prevalence of memory problems at 11% in a large group of COVID-19 patients eight months after the SARS-CoV-2 infection. Moreover, R Ferrucci and M Dini [24] found that COVID-19 survivors had deficits in verbal memory, attention, processing speed, and visuospatial after five months to one year.

The possible mechanism underlying such cognitive impairment is the effect of inflammatory cytokines triggered by SARS-CoV-2 infection, mainly interleukin-6 (IL-6) and tumor necrosis factor-α (TNF-α) [26]. These two cytokines can disrupt synaptic plasticity and alter hippocampal neurogenesis, which consequently causes cognitive decline [27]. The high neuroinvasive capability of the virus may add another contributing factor [28].

Cognitive decline was objectively determined in our patients who recovered from COVID-19 infection. EEG findings showed a significant increase in the absolute theta power and a decrease in absolute alpha power values in the post-COVID-19 group compared to the healthy control. Such a finding agrees with the Qeeg signature consistently reported during COVID-19 illness with increased amplitude in lower frequency bands and decreased amplitude in higher frequency bands [29–32].

A PET study also documented frontal lobe involvement in post-COVID-19 cognitive dysfunction, in which glucose hypometabolism in the frontoparietal cortex was correlated with the Montreal Cognitive Assessment (MoCA) score [19].

The frontoparietal is a fundamental brain region for short-term memory (e.g., working memory) [33], while the medial temporal region is for long-term memory [34]. In a systematic review carried out by D Shan, et al. [35], several functional and structural alterations have been described in the frontal, parietal, and temporal regions in COVID-19 survivors with memory impairments.

In addition to absolute power analyses, the level of Theta / Beta ratio was significantly higher in both central and parietal areas in the post-COVID-19 group than in the healthy controls. Theta/ Beta ratio is a commonly used marker of attentional capacity in variable neuropsychiatric disorders [17]. Moreover, the theta/beta ratio could distinguish cognitively normal elderly from MCI patients [36].

Besides, we found that patients who recovered from COVID-19 infection had significantly lower interhemispheric coherence for the frontal, central, and parietal areas in both alpha and beta bands. Low coherence appears to reflect diminished integrity of the structural connections between these brain regions [37]. Functional neuroimaging studies also revealed reduced brain functional connectivity in post-COVID-19 cognitively impaired subjects. Their study found hypo connectivity between dorsal and lateral prefrontal, somatosensory, and insula regions [38, 39].

In the current study, the need for oxygen support was significantly associated with memory and attentional deficits. The main mechanisms underlying hypoxia-related cognitive dysfunction are oxidative stress and neuronal injury [40]. In our study, lower interhemispheric coherence in the frontal regions in severe pneumonic patients who require oxygen support could explain this hypoxia-related cognitive profile. The current electrophysiological findings agree with the fMRI study by J Liu et al. [41], who found that the functional brain connectivity decreased significantly, particularly the frontoparietal network, after hypoxia exposure. However, the effects of hypoxia on functional connectivity depend on the hypoxic severity, which also goes with our results.

The main limitation of our study was the absence of a baseline cognitive assessment for the included patients before being infected with COVID-19. This would provide a more objective assessment of the impact of COVID-19 infection on cognitive function. Also, QEEG analyses for temporal and occipital areas were not evaluated because we were limited by the information we had in the database of healthy subjects. Having a control group provides more precise and reliable results. It is worth noting that non-exhaustive neuropsychological assessment might limit the generalizability of the results.

## Conclusion

Episodic memory deficit is evident in COVID-19 survivors with subjective cognitive complaints accompanied by lower inter-hemispheric coherence in frontal regions. These clinical and neurophysiological changes are associated with hypoxia and COVID-19 severity.

## Data Availability

The authors report that the data and materials that support the results or analyses presented in the current study will be freely available upon request from the corresponding author.
